# Fabrication of High Precision Silicon Spherical Microlens Arrays by Hot Embossing Process

**DOI:** 10.3390/mi13060899

**Published:** 2022-06-06

**Authors:** Quanquan Sun, Jiaxuan Tang, Lifeng Shen, Jie Lan, Zhenfeng Shen, Junfeng Xiao, Xiao Chen, Jianguo Zhang, Yu Wu, Jianfeng Xu, Xuefang Wang

**Affiliations:** 1 Shanghai Aerospace Control Technology Institute, Shanghai 201109, China; sunquanquan1992@163.com (Q.S.); fdchrist@126.com (L.S.); lan_jier@163.com (J.L.); little_fenger@sina.com (Z.S.); 2 State Key Laboratory of Digital Manufacturing Equipment & Technology, School of Mechanical Science and Engineering, Huazhong University of Science and Technology, Wuhan 430074, China; tjx@hust.edu.cn (J.T.); xiaojf@hust.edu.cn (J.X.); zhangjg@hust.edu.cn (J.Z.); mse_wuyu@hust.edu.cn (Y.W.); jfxu@hust.edu.cn (J.X.); 3 School of Mechanical Engineering, Hubei University of Technology, Wuhan 430068, China; xiaochen@hust.edu.cn

**Keywords:** silicon spherical microlens array, hot embossing, mold anti-sticking, ICP etching

## Abstract

In this paper, a high-precision, low-cost, batch processing nanoimprint method is proposed to process a spherical microlens array (MLA). The nanoimprint mold with high surface precision and low surface roughness was fabricated by single-point diamond turning. The anti-sticking treatment of the mold was carried out by perfluorooctyl phosphoric acid (PFOPA) liquid deposition. Through the orthogonal experiment of hot embossing with the treated mold and subsequent inductively coupled plasma (ICP) etching, the microstructure of MLA was transferred to the silicon substrate, with a root mean square error of 17.7 nm and a roughness of 12.1 nm Sa. The average fitted radius of the microlens array units is 406.145 µm, which is 1.54% different from the design radius.

## 1. Introduction

With the integration and development of multidisciplinary technologies such as optics, mechanics, and electricity, traditional optical devices have been unable to meet the high-performance requirements of contemporary high-precision optical equipment for optical components due to their high quantity, large volume, and poor anti-interference ability. The emergence of micro-optical elements provides a potential solution to this problem and allows for more application possibilities. The microlens array (MLA), consisting of many microlens units arranged in a certain way, is a symbol of micro-optical elements. Compared with traditional optical elements, the MLA can better adapt to the development trend of miniaturization, integration, array, and intelligence of optical elements.

The MLA has many optical functions, such as collimation, focusing, illumination, and imaging. Its excellent optical performance and small geometric footprint make it an important component for wide use in various fields, such as photoelectric devices [1,2], integrated micro-optics [3,4,5], imaging sensing [6,7,8], and so on. The fabrication technologies of MLAs include laser direct writing [9,10,11], overlay technology, grayscale lithography [12,13,14], photoresist (PR) reflow method [15,16], and nanoimprint lithography [17,18,19,20]. Direct writing technology has the advantages of high processing precision and high resolution, but its disadvantages of expensive equipment and low processing efficiency cannot be ignored. Overlay lithography can be used to make multistep relief structures, but the microstructure of continuous surfaces can only be approached by more steps. Although the principle of this technology is simple and mature, it needs multiple alignment exposure, development, and etching, resulting in difficultly guaranteeing the expected surface accuracy. Compared with overlay lithography, grayscale lithography requires only single exposure and development to obtain continuous microstructures. However, the complex coding principle and the difficulty of producing high-precision masks greatly limit its promotion and industrial application. The fabricated MLA through the PR reflow process has a smooth surface and low requirements for masks by forming spherical microstructures under liquid surface tension. Sadly, this method is not recommended for machining microlens arrays of different defined dimensions, due to the high time cost of establishing specific process parameters to control the microlens geometry at the end of the photoresist reflow step.

The nanoimprint lithography (NIL), proposed by Chou [21] in 1995, is dominated by two nanoimprint processes: thermal-NIL (hot embossing lithography, HEL) and UV-NIL [22], with high temperature or UV light for patterning, respectively. Compared with the latter, which needs a transparent mold or substrate and UV-curable intermediate polymer, the hot embossing process requires easier operating conditions. It has the advantages of a simple process, high resolution and efficiency, low cost, and good repeatability; it is also suitable for processing various microstructures [23,24], thus making it ever-evolved and widely used. This technology has no diffraction effect of traditional optical lithography technology and, thus, can achieve a resolution of up to 2.5 nm [25].

Many studies have been conducted on the fabrication methods of microlens arrays. Hua et al. [26] used femtosecond laser technology to process microlens arrays on silicon and achieved convex structures by removing silicon atoms with high-power laser beams. Femtosecond laser technology is simple and can work directly on the target material, but it is not suitable for processing large-area microstructures because of the low processing efficiency, as the laser beam is scanned point by point. The footprint of the area processed by Hua is typically only 100 × 100 µm^2^. Deng et al. [27] attempted to process large areas, but SEM images showed that the uniformity of array units was not satisfactory. Mukaida and Yan [28] investigated the process for fabricating silicon concave MLAs by the tool-servo driven segment turning method. However, the continuous cutting environment damages the diamond tool significantly, making it not applicable for mass production.

Compared with the above techniques, HEL provides a new idea for fabricating microlens arrays with high precision and high surface quality. Li et al. [29] fabricated an array of curable polymers, such as PMMA, with a lens size of 840 μm by hot embossing process. However, it is noted that this plastic lens array is easily scratched, and the time reliability is poor. Moreover, the material requires suitability for both the hot embossing process and optical function, thus limiting the variety of materials available. In this paper, we propose to transfer microlens arrays on polymers to rigid substrate materials such as silicon, glass, or other optical objects by inductively coupled plasma (ICP) etching, which ionizes SF_6_, C_4_F_8_, or other gases, followed by accelerating these plasmas by a bias voltage to vertically bombard the substrate material to enable large-area etching. We chose silicon as the substrate material on which to machine a 6×6 MLA with a lens width of 100 µm and a radius of curvature of 400 µm.

## 2. Materials and Methods

**Figure 1:**
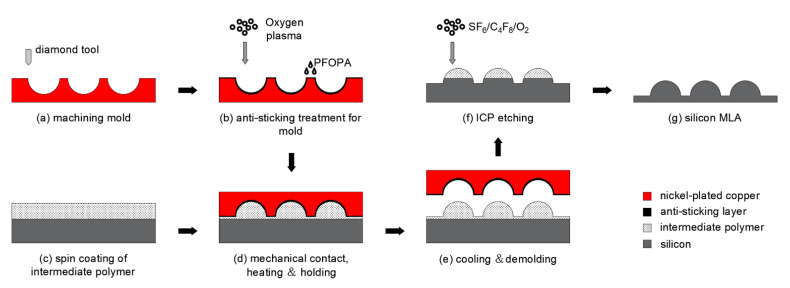
Schematic of the fabrication of MLA by HEL and ICP etching.

The entire experimental routine is as below (illustrated in Figure 1): first, the negative shape of the microlens array was processed on a nickel-plated mold, using single-point diamond turning (step a). Then the target microstructure was transferred to the intermediate polymer through hot embossing lithography (steps c, d, and e) and finally etched onto silicon by ICP etching (step f). To prevent adhesion between the polymer and the mold during demolding, a machined mold was necessarily treated with oxygen plasma and PFOPA (step b) to increase the surface contact angle before the HEL process.

### 2.1. Materials

The mold material used in the experiment was a 2-inch copper chip coated with an 80 μm nickel layer (nickel–phosphorus alloy with 87.3% nickel and 12.7% phosphorus content). MR-I T85-5.0, used as the intermediate polymer and sourced from Micro Resist Technology (Berlin, Germany), has a glass transition temperature of 85 °C. Its good thermoplasticity and stable chemical properties that allow for the perfect replication of microstructures on the mold. MLA material selected silicon, purchased from RDMICRO company (Suzhou, China), was cut into 1/4 samples from 2-inch wafer for experiments. The silicon we used is N-type, with a crystal orientation of <111>, polished on both sides and 200 μm thick. Perfluorooctyl phosphonic acid (PFOPA) and the corresponding solvent methyl tert-butyl ether (MTBE), used for anti-sticking treatment, must be attentively operated due to their toxicity. The untreated mold and all silicon samples were cleaned ultrasonically with isopropyl alcohol, anhydrous ethanol, and deionized water, respectively, for 5 min and baked at 100 °C for 10 min before the experiment.

### 2.2. Experimental Equipment

The opposite microstructure of MLA on the mold was fabricated by an ultra-precision CNC contouring machine (Precitech, Nanoform^®^ X, Keene, NH, USA), configured with four axes to produce spherical, aspherical, and freeform surfaces of up to 440 mm in diameter. The hot embossing process was operated on the EITRE^®^ Nano Imprint Lithography System (Obducat, EITRE^®^ 3, Lund, Sweden), and the ICP etcher used was Plasmalab System 100 ICP 180 (Oxford, Yatton, UK).

### 2.3. Methods

#### 2.3.1. Single-Point Diamond Turning Method for Making Mold of MLA

**Figure 2:**
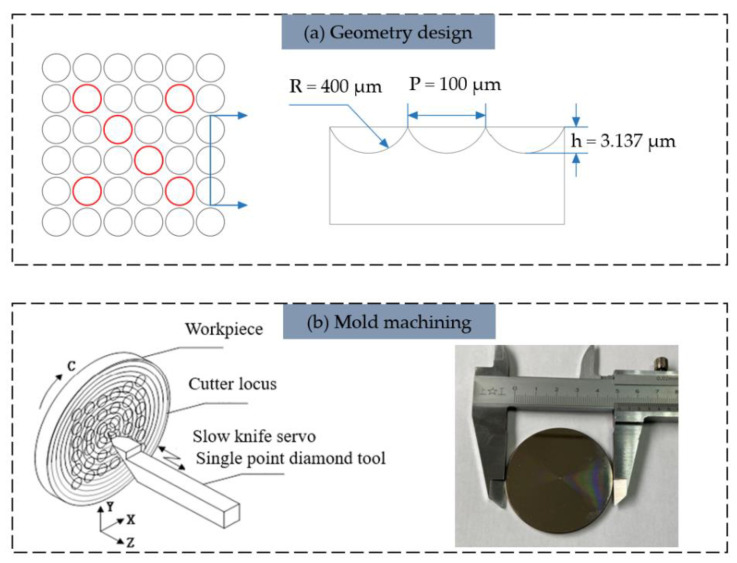
Mold design and machining: (**a**) geometry design of MLA structure, and (**b**) mold machining and schematic diagram of cutting.

In this experiment, considering the processing efficiency, production cost and service life, the single-point diamond turning (SPDT) [30] method was determined to process the rigid molds of MLA. Slow tool servo (STS) machining with XZC 3-axis linkage was performed on the ultra-precision lathe. A particular single crystal diamond tool carved out the negative structure of MLA, following a spiral toolpath that was generated by controlling X and C axes in a discrete mode of equal arc length (indicated in Figure 2b). The cutting conditions and tool parameters are summarized in Table 1. The toolpath data were automatically generated by the lathe supportive software DIFFSYS based on manual settings, and then the specific document containing process information was copied into the machine tool equipment. Rough tool setting, dynamic balance adjustment, and trial cutting tool settings were completed successively. The aforesaid preparations consumed 2 h, and the final cutting schedule was 656 s. The MLA mold was fabricated by the above routine with designed dimensions of 400 μm radius of curvature, 100 μm bottom width, and 3.137 μm height (shown in Figure 2a). Six units marked in red from the MLA were selected for measurement after each process step. All subsequent dimensional characterizations of array structure are averaged from the corresponding inspection data at the same locations.

#### 2.3.2. Anti-Sticking Treatment of Nickel Mold

The demolding step in NIL has a significant influence on the precision of final microstructure. The surface of mold without anti-sticking treatment will stick with the intermediate polymer and be contaminated [31,32,33].

Differing from the conventional physical deposition that uses liquid or gaseous perfluorooctyl trichlorosilane (F13-TCS), PFOPA and oxygen plasma have been explored as a new method of anti-sticking. The anti-sticking treatment process of PFOPA is as follows: the plasma cleaner (Diener Pico, Ebhausen, Germany) ionized oxygen under 50 W/50 Pa conditions to generate plasma, impacting the nickel mold for 5 min, enabling the mold surface to form a layer of nickel oxide. A total of 1 g of PFOPA was dissolved in 150 mL of MTBE solution inside an ultrasonic cleaner (KQ-600KDE, Kunshan, China) to enable better dissolution of both. The mold was immersed in the solution for 4 h within 30 min after oxygen plasma treatment and dried subsequently. After anti-sticking treatment, the drop shape analyzer (Kruss, DSA25, Hamburg, Germany) was used to measure the contact angle of the mold surface. If the contact angle is greater than 90°, the mold demonstrates anti-sticking property and will not adhere with polymer during the demolding step.

#### 2.3.3. Orthogonal Experiment of Hot Embossing Process

**Figure 3:**
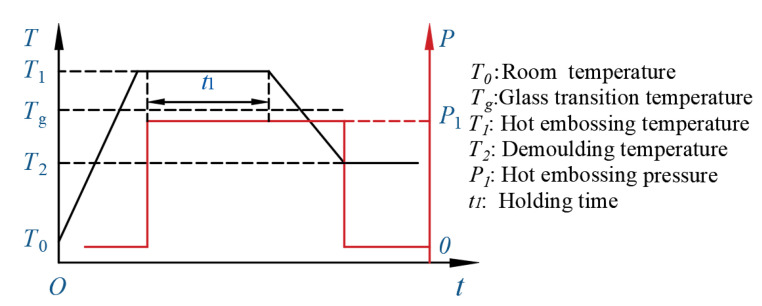
Temperature and pressure change curve with time in HEL.

The hot embossing lithography (HEL) process mainly includes mechanical contact, heating, holding, cooling, and demolding, and it is simpler than traditional photolithography. The intermediate polymer used is generally thermoplastic resin or resist in HEL. During the thermal curing process, thermoplastic polymer was heated to a temperature T1, above its glass transition temperature Tg, and applied pressure P1 for a period of time t1. After cooling down to a release temperature T2 below Tg, the pressure was removed, and the mold was released. The relationship between temperature and pressure with time in hot embossing is shown in Figure 3.

The main factors that affect microlens reproduction rate (the percentage of the maximum height of the imprinted microstructure on the polymer and the height of the mold microstructure) are temperature, holding time, and imprint pressure. According to experience and the type of intermediate polymer, different levels of the influencing parameters were selected in the orthogonal experiment. In this experiment, the L_9_(3^4^) orthogonal table was selected for experimental study, and it was suitable for the hot embossing experiment with three factors and three levels, as shown in Table 2.

#### 2.3.4. ICP Etching Experiment

During ICP etching [34,35,36], a top RF source (ICP power) ionizes the etching gas into plasma, and the electromagnetic field applied by a bottom RF source (RF power) causes the resulting plasma to bombard the sample surface vertically downward for enabling the removal of surface material both chemically and physically. In order to obtain the microstructure with high precision and surface quality, it is necessary to optimize the process parameters, including chamber pressure, RF power, ICP power, etching time, etching gas type, proportion, and flow rate.

**Figure 4:**
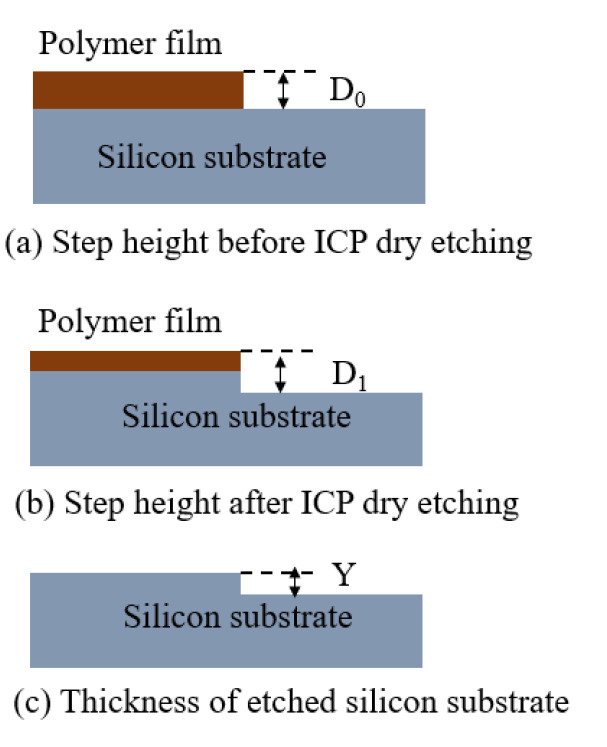
Schematic diagram of selectivity calculation.

For high fidelity transfer of microstructure on the intermediate polymer to the silicon substrate, the ICP etching parameters with the selectivity (ratio of etching rate of substrate material to the etching rate of intermediate polymer) close to 1:1 should be selected for pattern transfer. There will be a height difference between the etched microstructure on the silicon substrate and original on the polymer with other selectivities. The polymer can be etched with O_2_, generating waste gas CO_2_ and H_2_O. SF_6_ and C_4_F_8_ etched silicon substrates, where the former generated SiF_4_ gas with silicon and the latter reduced lateral etching and protected sidewalls. The selectivity is calculated by the following formula (illustrated in Figure 4):

(1) $$q=\frac{Y}{D_{0}-D_{1}+Y}$$

### 2.4. Characterization

The surface morphology of MLA microstructures was observed by a metallographic microscope (Zeiss, Axiocam 208 color, Jena, Germany). A white light interferometer (Zygo, NewView 9000, Middlefield, CT, USA) was used to obtain the 3D morphology and surface roughness of the microstructures. A stylus profilometer (Bruker DektakXT, Billerica, MA, USA) was utilized to extract cross-section profiles of the microstructures.

## 3. Results and Discussions

### 3.1. Inspection Results of Hot Embossing Mold

**Figure 5:**
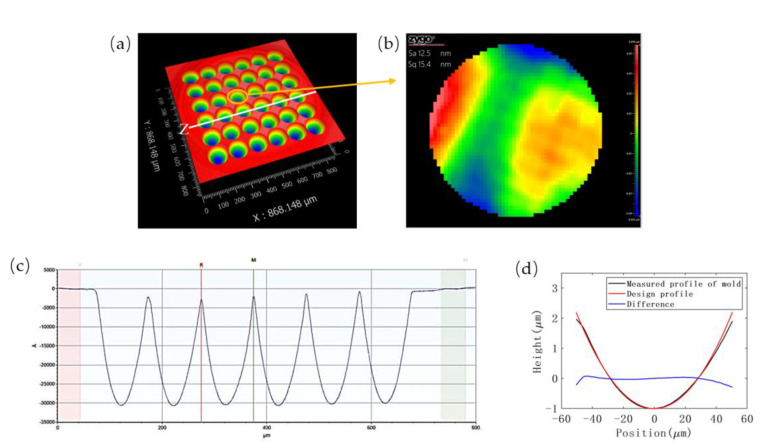
The MLA characterization of mold: (**a**) 3D morphology of the mold, (**b**) roughness inspection image of the marked unit, (**c**) the profile of Z section, and (**d**) comparison of measured surface profile and design profile.

The concave microstructure of MLA was successfully fabricated by single-point diamond turning method and characterized by using the white light interferometer and stylus profilometer. The 3D morphology in Figure 5a and cross-section profile in Figure 5c display the negative geometry of MLA. The contour of the machining unit fits well with the ideal curve, as shown in Figure 5d, plotted by the software MATLAB R2019a. These measurement results demonstrate good uniformity and high surface quality of the resulting mold, with a root mean square error (RMSE) of 15.5 nm and a low surface roughness average of 11.1 nm. A roughness inspection result of one unit is shown in Figure 5b. The fitted radius of the machining units on the mold is 403.139 μm, which is only 0.78% different from the design dimension of 400 μm.

### 3.2. Effect of Anti-Sticking Treatment of Nickel Mold

Three anti-sticking treatments were investigated in this experiment: gas-phase and liquid-phase treatment using F13-TCS; and liquid-phase treatment using PFOPA. The change in contact angle before and after the anti-sticking treatments was measured to characterize the anti-sticking property of the mold. From the average results after repeated measurements, the contact angle of the mold surface without anti-sticking treatment is only about 60°, which is hydrophilic. However, after these anti-sticking processes described, it increases to >100°, verifying the hydrophobic nature and resistance to polymers.

**Figure 6:**
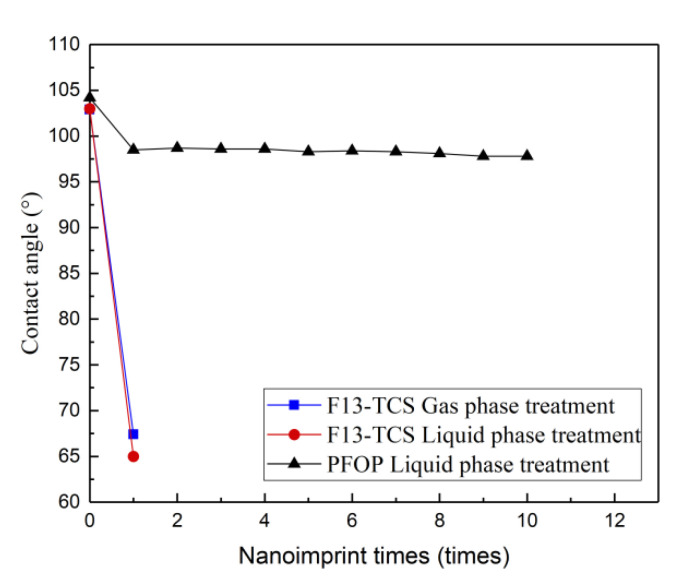
The relationship between the number of impressions and the contact angle of different anti-sticking treatments.

**Figure 7:**
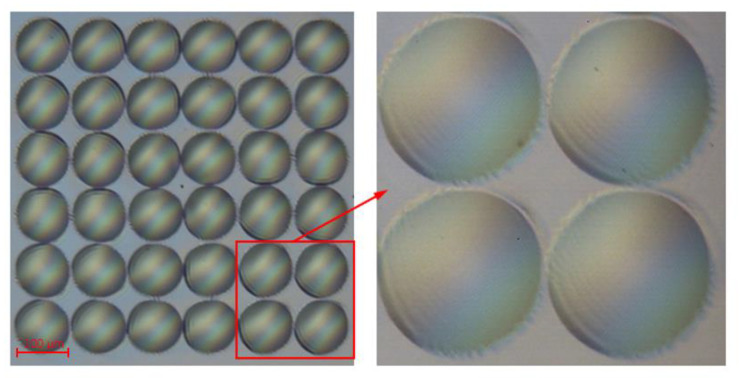
The micrograph of the mold after 80 times of use.

The experimental data clearly reveal that the anti-sticking property obtained by F13-TCS treatment is disposable, related to the weak bonding between F13-TCS film and the mold surface, resulting from the physical deposition of the film on the mold surface. The mold treated with PFOPA can preserve hydrophobic over time without repetitive operations. The variation of contact angle with the number of uses is shown in Figure 6, below. During the anti-sticking treatment with PFOPA, a layer of nickel oxide is generated on the surface of the mold after oxygen plasma treatment. Nickel oxide reacts with PFOPA, chemically forming a self-assembled monolayer (SAM) [37,38] on the mold surface that is responsible for the hydrophilic nature of the mold. The anti-sticking layer can attach stably to mold surface even under high temperature and pressure during HEL, contributing to the reusability of the mold. Since the anti-sticking layer is only a single molecular film, with a thickness normally thinner than 2.5 nm [39], which is significantly less than the design size of the MLA; thus, its effect on the surface morphology is out of consideration. The micrograph in Figure 7 obtained after multiple utilization reveals a spotless surface of the mold with no residual polymer. 

In addition, the mold after anti-sticking treatment has a certain self-cleaning effect. When dust or other particles accidentally fall on the mold, a piece of IPS film is subjected to the hot embossing process with the mold and carries away the contaminants during detachment.

### 3.3. Results and Analysis of Hot Embossing Experiments

**Figure 8:**
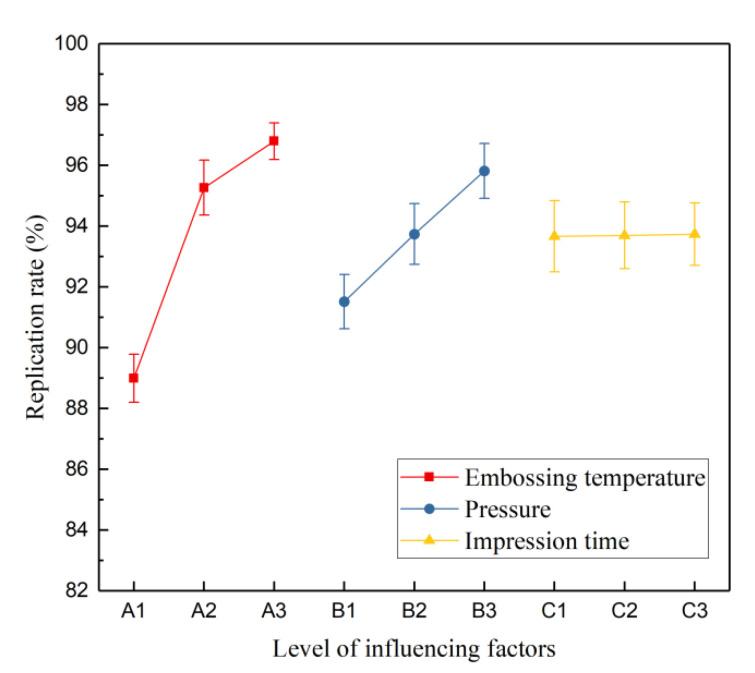
The relationship between replication rate and different levels of various factors.

The microstructure pattern on the mold was successfully transferred to thermoplastic polymer, and nine groups of hot embossing experiments were carried out with the nanoimprint instrument. The experimental data, as shown in Table 3, adopt the method of the range analysis and focus on the replication effect of positive patterns on the polymer transferred from mold. The replication effect (or replication rate), regarded as the transfer accuracy of HEL, is characterized by the percentage of the average height of the microlens units on the polymer to the sag height of the mold microstructure. M1, M2, and M3 denote the effects of different levels of each factor on the transfer accuracy, respectively, and R indicates the ranges of corresponding factors. The relationship between different levels of each factor and replication rates is exhibited in Figure 8.

**Figure 9:**
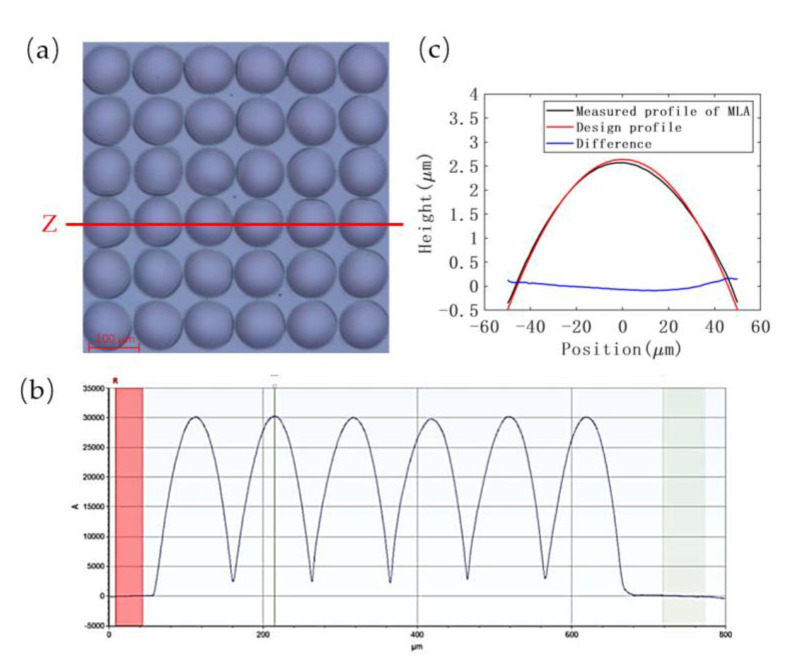
The MLA characterization after HEL: (**a**) micrograph of imprinting result, (**b**) the profile of Z section, and (**c**) comparison of the result of HEL and ideal structure contour.

The order of temperature, pressure, and embossing time is arranged by the magnitude of ranges, as the higher range indicates that the corresponding factor affects the reproduction effect more significantly. Figure 8 visualizes that the increase in temperature and pressure improves the replication accuracy, different from the embossing time, which has no effect. Based on comparing the average replication rates, M1–M3, of different levels in Table 3, the theoretically optimal combination was A_3_B_3_C_3_. However, the range R3 of the embossing time is almost equal to 0. This indicates that, for the hot embossing step in this process, when the holding time reaches 180 s, the embossing is already completed, and it is no longer significant to continue increasing the embossing time. A long embossing time barely increases the replication effect, but it delays the experimental schedule. After consideration, the combination of A_3_B_3_C_2_ (180 °C, 32 bar, and 240 s) can achieve a 97% replication rate, indeed determined as the final parameter combination. The height of patterned microlens structure on the intermediate polymer is 3.006 μm, as shown in Figure 9a,b for the test results. The measured profile has a gap at the peak compared to the ideal profile (shown in Figure 9c), caused by the height difference from incomplete filling of the polymer during HEL process. This height difference will be compensated by adjusting the selectivity in the subsequent ICP etching.

### 3.4. Results and Analysis of ICP Etching Experiment

ICP etching results depend on the process parameters, such as chamber pressure, RF power, ICP power, etching time, etching gas type, proportion, and flow rate. In the experiment, 18 series were performed to investigate the effect of RF power and gas flow rate on the selectivity during ICP etching with constant pressure and ICP power. The experimental parameters and results are listed in Table 4 below.

**Figure 10:**
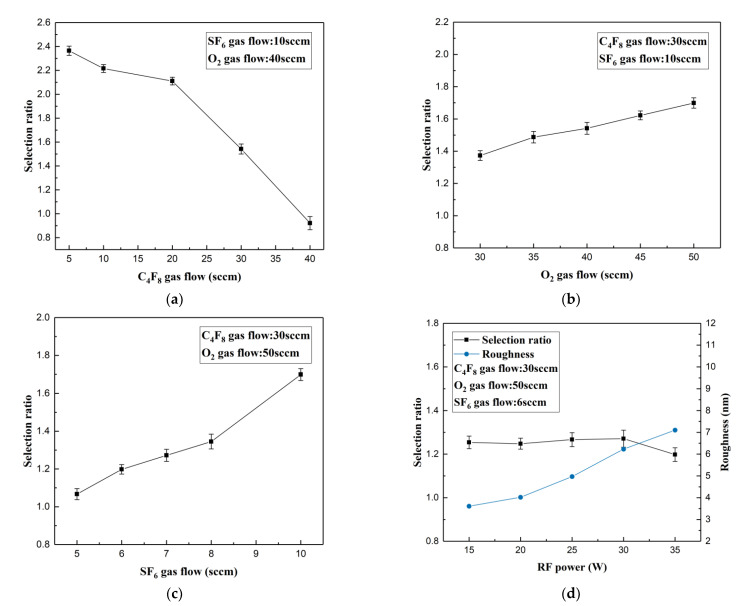
The influence of (**a**) C_4_F_8_, (**b**) O_2_, (**c**) SF_6_, and (**d**) RF power on selectivity.

The analyzed results are visualized in Figure 10. These results reveal that the selectivity increases with O_2_ and SF_6_, with an opposite contribution of C_4_F_8_. C_4_F_8_ primarily forms a passivation layer (CF_2_ polymer chain) on the surface of the silicon substrate during etching process. F^−^ ions ionized from SF_6_ can contact and remove silicon molecules only after consuming the protective layer, and these two steps are continuously cycled. Thus, the flow rates of SF_6_ and C_4_F_8_ crucially master the etching rate of silicon. It is also clearly concluded that SF_6_ plays a positive role, while the latter plays a negative role. The increase in O_2_ accelerates the removal of intermediate polymer on the substrate surface, but also rapidly depletes the C element in C_4_F_8_, which reduces the etching rate of the silicon. The incidence rate of etched ions increases with RF frequency, enhancing physical etching. This effect improves the etching rate, but also damages the surface of newly etched microstructure and reduces its surface roughness.

**Figure 11:**
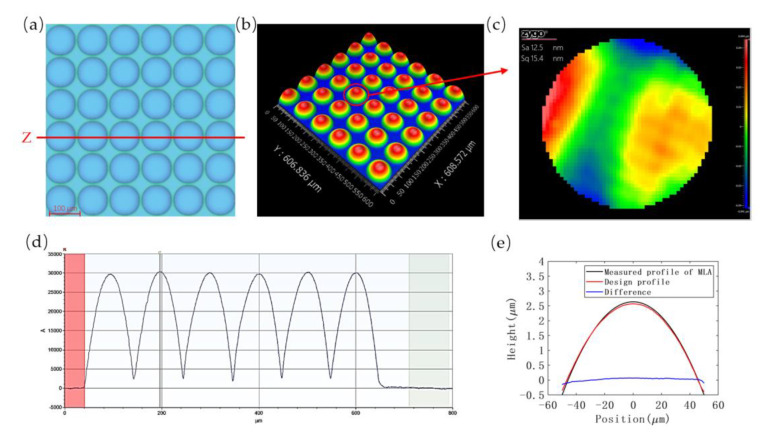
The silicon MLA characterization after ICP etching: (**a**) surface morphology of silicon MLA, (**b**) 3D view of the MLA, (**c**) roughness inspection image of the marked unit, (**d**) the profile of Z section, and (**e**) comparison of final result and ideal profile.

The selectivity needs to be appropriately larger than 1:1, as the previous HEL process resulted in a smaller height of embossed structure on the polymer than desired dimension. Meanwhile, according to the experiment, it is observed that selectivity decreases as time passes during etching. Weighing the roughness and etching time, the parameters of the 17th group were finally selected for pattern transfer, and the etching process continued for 23 min. The surface profile and micrograph of fabricated silicon MLAs are displayed in Figure 11a,b,d. The root mean square error of the surface profile compared with design profile is 17.7 nm and the surface roughness average of the microlens array is 12.1 nm Sa. The average fitted radius of the microlens’ array units is 406.145 µm, which is 1.54% different from the design radius. The theoretical focal length (*f*) of the microlens is 163.8 µm, as calculated by the following formula:

(2) $$f=\frac{R}{n-1}$$

where *R* and *n* are the curvature radius of the lens and the refractive index of silicon (*n* = 3.48). This microlens array is available for mid-wave infrared (3–5 µm) optical systems.

## 4. Conclusions

In summary, the hot embossing process, followed by ICP etching, was demonstrated to be a viable method to fabricate microlens arrays on silicon. A high-precision and high-surface-quality nanoimprint mold with a surface roughness of 11.1 nm Sa was fabricated by single-point diamond turning. Then the HEL parameters were optimized to a combination with the temperature of 180 °C, pressure of 32 bar, and an embossing time of 240 s by orthogonal experiment. Finally, the MLA microstructure was successfully transferred to the silicon substrate by ICP etching. In order to ensure the smooth demolding and reusability of the mold, it is necessary to apply an anti-sticking treatment to the mold.

In the anti-sticking treatment process, we used PFOPA and plasma oxidation instead of F13-TCS. Compared with the latter, PFOPA can maintain a contact angle of >95° under the cycle of continuous heating, pressurization, cooling, and depressurization due to the formation of chemical bonding, thus avoiding the dilemma of repetitive anti-sticking treatments. The results of the orthogonal experiment prove that temperature and pressure are significantly more influential than embossing time, thus indicating that a long duration only delays the embossing efficiency. In the ICP etching process, the etching rate of silicon is controlled mainly by SF_6_ and C_4_F_8_ to adjust the selectivity, and a low roughness requires proper RF power. O_2_ accelerates the removal of polymers, but it also apparently affects silicon.

The silicon spherical microlens array fabricated by the hot embossing process and subsequent ICP etching has a surface roughness of 12.1 nm Sa and a RMSE of 17.7 nm Sq., differing from the design radius by 1.54%, which provides a new potential for manufacturing MLA. This scheme can be used for mass production of microlens arrays in the future, but the drawback of progressively more difficult mold fabrication as the number of array units increases needs further research. Future work includes testing the optical properties of silicon microlens arrays and increasing the number of array units.

## Figures and Tables

**Table 1:** Cutting conditions.

Cutting Conditions	Value
Workpiece material	Nickel–phosphorus alloy
Tool rake angle	0°
Tool relief angle	15°
Tool nose radius	0.1 mm
Depth of cut	from 2 to 5 μm
*X*-axis interpolation increments per revolution	0.001 mm
C-axis interpolation arc length increment	0.001 mm
Interpolation time interval	2 ms

**Table 2:** Factors and levels of hot embossing orthogonal experiment.

Level	A: Embossing Temperature (T1)/°C	B: Embossing Pressure (P1)/bar	C: Embossing Time (t1)/s
1	140	20	180
2	160	26	240
3	180	32	300

**Table 3:** Orthogonal experiment table of hot embossing process.

Number	Experimental Factors			Replication Effect
	Embossing Temperature (T1)/°C	Embossing Pressure (P1)/bar	Embossing Time (t1)/s	
1	140	20	180	85.8%
2	140	26	240	89.6%
3	140	32	300	91.6%
4	160	20	240	93.5%
5	160	26	300	94.4%
6	160	32	180	97.8%
7	180	20	300	95.2%
8	180	26	180	97.3%
9	180	32	240	98.0%
M1	89.0%	91.5%	93.7%	
M2	95.2%	93.8%	93.7%	
M3	96.8%	95.8%	93.7%	
R	0.239	0.132	0.002	

**Table 4:** Parameters and results of selectivity adjustment experiments.

Number	Chamber Pressure (mTorr)	ICP Power (W)	RF Power (W)	Gas Flow (Sccm)			Selectivity
				C_4_F_8_	SF_6_	O_2_	
1	10	800	35	30	10	40	1.542:1
2	10	800	35	30	10	50	1.699:1
3	10	800	35	30	10	45	1.622:1
4	10	800	35	30	6	50	1.198:1
5	10	800	35	20	10	40	2.111:1
6	10	800	35	10	10	40	2.216:1
7	10	800	35	30	6	50	1.207:1
8	10	800	35	30	5	50	1.067:1
9	10	800	35	30	7	50	1.272:1
10	10	800	35	40	10	40	0.922:1
11	10	800	35	5	10	40	2.365:1
12	10	800	35	30	10	35	1.487:1
13	10	800	35	30	10	30	1.373:1
14	10	800	35	30	8	50	1.345:1
15	10	800	30	30	6	50	1.271:1
16	10	800	25	30	6	50	1.267:1
17	10	800	20	30	6	50	1.248:1
18	10	800	15	30	6	50	1.254:1

## Data Availability

Data sharing is not applicable to this article.

## References

[1] Liu X.Q., Yu L., Yang S.N., Chen Q.D., Wang L., Juodkazis S., Sun H.B. (2019). Optical Nanofabrication of Concave Microlens Arrays. Laser Photon. Rev..

[2] Qu Y., Kim J., Coburn C., Forrest S.R. (2018). Efficient, Nonintrusive Outcoupling in Organic Light Emitting Devices Using Embedded Microlens Arrays. ACS Photonics.

[3] Petsch S., Schuhladen S., Dreesen L., Zappe H. (2016). The engineered eyeball, a tunable imaging system using soft-matter micro-optics. Light Sci. Appl..

[4] Lee G.J., Yoo Y.J., Song Y.M. (2018). Recent advances in imaging systems and photonic nanostructures inspired by insect eye geometry. Appl. Spectrosc. Rev..

[5] Wu D., Xu J., Niu L.G., Wu S.Z., Midorikawa K., Sugioka K. (2015). In-channel integration of designable microoptical devices using flat scaffold-supported femtosecond-laser microfabrication for coupling-free optofluidic cell counting. Light Sci. Appl..

[6] Sieler M., Schreiber P., Dannberg P., Bräuer A. (2011). Design and realization of an ultra-slim array projector. Proceedings of the 17th Microopics Conference (MOC).

[7] Kim S.M., Kim H., Kang S. (2006). Development of an ultraviolet imprinting process for integrating a microlens array onto an image sensor. Opt. Lett..

[8] Urey H., Powell K.D. (2005). Microlens-array-based exit-pupil expander for full-color displays. Appl. Opt..

[9] Yu W., Yuan X., Ngo N., Que W., Cheong W., Koudriachov V. (2002). Single-step fabrication of continuous surface relief micro-optical elements in hybrid sol-gel glass by laser direct writing. Opt Express.

[10] Fu Y., Bryan N.K. (2002). Semiconductor microlenses fabricated by one-step focused ion beam direct writing. IEEE Trans. Semicond. Manuf..

[11] Gissibl T., Thiele S., Herkommer A., Giessen H. (2016). Two-photon direct laser writing of ultracompact multi-lens objectives. Nat. Photonics.

[12] Waits C.M., Morgan B., Kastantin M., Ghodssi R. (2004). Microfabrication of 3D silicon MEMS structures using gray-scale lithography and deep reactive ion etching. Sens. Actuator A-Phys..

[13] Waits C.M., Ghodssi R., Ervin M.H., Dubey M. (2001). MEMS-based gray-scale lithography. Semicond. Device Res. Symp. ISDRS—Proc..

[14] Totsu K., Esashi M. (2005). Gray-scale photolithography using maskless exposure system. J. Vac. Sci. Technol. B.

[15] Chung C.K., Hong Y.Z. (2007). Fabrication and analysis of the reflowed microlens arrays using JSR THB-130 N photoresist with different heat treatments. Microsyst. Technol..

[16] Grigaliūnas V., Lazauskas A., Jucius D., Viržonis D., Abakevičienė B., Smetona S., Tamulevičius S. (2016). Microlens fabrication by 3D electron beam lithography combined with thermal reflow technique. Microelectron. Eng..

[17] Kim Y.K., Ju J.H., Kim S.M. (2018). Replication of a glass microlens array using a vitreous carbon mold. Opt. Express.

[18] Chang C.Y., Yu C.H. (2015). A basic experimental study of ultrasonic assisted hot embossing process for rapid fabrication of microlens arrays. J. Micromech. Microeng..

[19] Chang C.Y., Yang S.Y., Huang L.S., Chang J.H. (2006). Fabrication of plastic microlens array using gas-assisted micro-hot-embossing with a silicon mold. Infrared Phys. Technol..

[20] Schulz H., Wissen M., Bogdanski N., Scheer H.C., Mattes K., Friedrich C. (2005). Choice of the molecular weight of an imprint polymer for hot embossing lithography. Microelectron. Eng..

[21] Chou S.Y., Krauss P.R., Renstrom P.J. (1995). Imprint of sub-25 nm vias and trenches in polymers. Appl. Phys. Lett..

[22] Asif M.H., Graczyk M., Heidari B., Maximov I. (2022). Comparison of UV-curable materials for high-resolution polymer nanoimprint stamps. Micro Nano Eng..

[23] Graczyk M., Cattoni A., Rösner B., Seniutinas G., Löfstrand A., Kvennefors A., Maximov I. (2018). Nanoimprint stamps with ultra-high resolution: Optimal fabrication techniques. Microelectron. Eng..

[24] Jia Z., Choi J., Park S. (2018). Selection of UV-resins for nanostructured molds for thermal-NIL. Nanotechnology.

[25] Hua F., Sun Y., Gaur A., Meitl M.A., Bilhaut L., Rotkina L., Shim A. (2004). Polymer imprint lithography with molecular-scale resolution. Nano Lett..

[26] Hua J.G., Ren H., Jia A., Tian Z.N., Wang L., Juodkazis S., Chen Q., Sun H.B. (2020). Convex silica microlens arrays via femtosecond laser writing. Opt. Lett..

[27] Deng Z., Yang Q., Chen F., Meng X., Bian H., Yong J., Chan C., Hou X. (2015). Fabrication of large-area concave microlens array on silicon by femtosecond laser micromachining. Opt. Lett..

[28] Mukaida M., Yan J. (2017). Fabrication of hexagonal microlens arrays on single-crystal silicon using the tool-servo driven segment turning method. Micromachines.

[29] Li Y., Li K., Gong F. (2021). Fabrication and Optical Characterization of Polymeric Aspherical Microlens Array Using Hot Embossing Technology. Appl. Sci..

[30] Zhang G., Dai Y., Lai Z. (2021). A novel force-based two-dimensional tool centre error identification method in single-point diamond turning. Precis. Eng..

[31] Yang Z.H., Chiu C.Y., Yang J.T., Yeh J.A. (2009). Investigation and application of an ultrahydrophobic hybrid-structured surface with anti-sticking character. J. Micromech. Microeng..

[32] Keil M., Beck M., Ling T.G.I., Graczyk M., Montelius L., Heidari B. (2005). Development and characterization of silane antisticking layers on nickel-based stamps designed for nanoimprint lithography. J. Vac. Sci. Technol. B.

[33] Padeste C., Bellini S., Siewert D., Schift H. (2014). Anti-sticking layers for nickel-based nanoreplication tools. Microelectron. Eng..

[34] Sökmen Ü., Balke M., Stranz A., Fündling S., Peiner E., Wehmann H.H., Waag A. (2009). ICP cryogenic dry etching for shallow and deep etching in silicon. Smart Sensors, Actuators, and MEMS IV.

[35] Duanmu Q., Zhang A., Wang G., Gao Y., Li Y., Jiang D., Tian J., Ma Z.C., Jin G.F., Chen X.Y. (2004). Silicon microhole array prepared by ICP. MEMS/MOEMS Technologies and Applications II, Photonics Asia, Beijing, China, 8–11 November 2004.

[36] Henry M.D. (2010). ICP Etching of Silicon for Micro and Nanoscale Devices.

[37] Gawalt E.S., Avaltroni M.J., Koch N., Schwartz J. (2001). Self-assembly and bonding of alkanephosphonic acids on the native oxide surface of titanium. Langmuir.

[38] Keil M., Beck M., Frennesson G., Theander E., Bolmsjö E., Montelius L., Heidari B. (2004). Process development and characterization of antisticking layers on nickel-based stamps designed for nanoimprint lithography. J. Vac. Sci. Technol. B.

[39] Lee M.J., Cho S.U., Lee S.M., Kim C.S., Jeong M.Y. (2012). Enhanced uniformity and durability of antisticking layer in imprinting stamps. Appl. Surf. Sci..

